# Geographical variation and predictors of zero utilization for a standard maternal continuum of care among women in Ethiopia: a spatial and geographically weighted regression analysis

**DOI:** 10.1186/s12884-021-04364-6

**Published:** 2022-01-28

**Authors:** Mequannent Sharew Melaku, Agazhe Aemro, Setognal Birara Aychiluhm, Amare Muche, Gizachew Kassahun Bizuneh, Shimels Derso Kebede

**Affiliations:** 1 Department of Health Informatics, Institute of Public Health, College of Medicine and Health Sciences, University of Gondar, Gondar, Ethiopia; 2 Department of Medical Nursing, School of Nursing, College of Medicine and Health Sciences, University of Gondar, Gondar, Ethiopia; 3 Department of Public Health, College of Medicine and Health Sciences, Samara University, Samara, Ethiopia; 4 Department of Epidemiology and Biostatistics, School of Public Health, College of Medicine and Health Sciences, Wollo University, Dessie, Ethiopia; 5 Department of Pharmacognosy, School of Pharmacy, College of Medicine and Health Sciences, University of Gondar, Gondar, Ethiopia; 6 Department of Health Informatics, School of Public Health, College of Medicine and Health Science, Wollo University, Dessie, Ethiopia

**Keywords:** Zero utilization for maternal continuum of care, Spatial analysis, Geographically weighted regression

## Abstract

**Background:**

Maintaining and effectively utilizing maternal continuum of care could save an estimated 860,000 additional mothers and newborn lives each year. In Ethiopia, the number of maternal and neonatal deaths occurred during pregnancy, childbirth, and the postpartum period was very high. It is indisputable that area-based heterogeneity of zero utilization for a standard maternal continuum of care is critical to improve maternal and child health interventions. However, none of the previous studies explored the spatial distribution of zero utilization for maternal continuum of care. Hence, this study was aimed to explore geographical variation and predictors of zero utilization for a standard maternal continuum of care among women in Ethiopia.

**Methods:**

A total of 4178 women who gave birth five years preceding the 2016 Ethiopian demographic and health survey were included. ArcGIS version 10.7, SaT Scan version 9.6, and GWR version 4.0 Software was used to handle mapping, hotspot, ordinary least square, Bernoulli model analysis, and to model spatial relationships. Finally, a statistical decision was made at a *p*-value< 0.05 and at 95% confidence interval.

**Main findings:**

The proportion of mothers who had zero utilization of a standard maternal continuum of care was 48.8% (95% CI: 47.3–50.4). Hot spot (high risk) regions for zero utilization of maternal continuum of care was detected in Afder, Warder, Korahe and Gode Zones of Somali region and West Arsi Zone of Oromia region. Respondents who had poor wealth index, uneducated mothers, and mothers who declared distance as a big problem could increase zero utilization of maternal continuum of care by 0.24, 0.27, and 0.1 times.

**Conclusion:**

Five women out of ten could not utilize any components of a standard maternal continuum of care. Hot spot (high risk) areas was detected in Afder, Warder, Korahe and Gode Zones of Somali region and West Arsi Zone of Oromia region. Poor wealth index, uneducated mothers, and mothers who declare distance as a big problem were factors significantly associated with zero utilization of maternal continuum of care. Thus, geographical based intervention could be held to curve the high prevalence of zero utilization of maternal continuum of care.

## Background

Every day, over 800 mothers die as a result of pregnancy and delivery-related health complications, with 94% of these deaths occurring in low-resource settings. Every year, around 2.9 million infants and 265,000 mothers die as a result of complications during pregnancy and delivery. Over half of all maternal and neonatal deaths occurred in Sub-Saharan African (SSA) countries [1, 2]. Ethiopia is currently identified among countries with highest maternal mortality ratio (MMR) and neonatal deaths [3].

Providing continuous care during the reproductive, maternal, newborn, and child health (RMNCH) period is an important strategy to reduce maternal and neonatal mortality. The World Health Organization (WHO) has called for a “continuum of care” for reproductive, maternal, neonatal, and child health services to improve maternal and newborn health [4, 5]. The RMNCH services encompasses integrated service delivery from pre-pregnancy to delivery, as well as the immediate postnatal and childhood periods [6, 7]. It also recognizes that safe childbirth is critical to the health of the woman and the newborn child, as well as an essential step towards a sound childhood and a productive life. Thus, if maternal continuum of care is maintained and effectively utilized an estimated 860,000 additional mothers and newborn lives could be saved each year [8, 9]. Skilled care before, during, and after birth reduces the risk of death or disability for both the mother and the baby. On the other hand, a lack of care at any level of the RMNCH period is associated with poor maternal and child health outcomes [10–12].

Despite the fact that maternal health services are available in all kebeles, they are underutilized. As part of WHO advocacy Ethiopia is providing integrated service for mothers and children from pre-pregnancy to childhood periods. However, the use of services for maternal continuity of care is still doubtful. The Ethiopian demographic and health survey (EDHS) reported that antenatal coverage, institutional delivery, and postnatal care were (64%), (28%), and (17%) respectively [3]. This indicated that a significant number of women could not receive any type of care during the period of maternal continuum of care. Several studies conducted across the world have identified several factors associated with the completion of maternal continuum of care [13–16].

Improving maternal health is one of the targets of the third Sustainable Development Goals (SDGs) [17]. This could not possible without the participation of women in any type of care stated under the continuum of maternal health service. Different strategies, policies, and programs have been attempted to improve safe motherhood at global, regional, and national level [13, 18–21].

Some studies were conducted in Ethiopia on the spatial distribution of antenatal care, skilled birth attendance and postnatal care separately [22–27]. Instead of looking non utilization of maternal health services separately, this study was aimed to examine non-utilization of any components of maternal continuum of care, including four or more ANC visit, delivery assisted by SBA, and PNC within 48 h. Since, a woman who could not use any component of a standard maternal continuum of care is highly marginalized, this study could provide valuable insight for maternal health service uptake.

It is indisputable that area-based heterogeneity of zero utilization for a standard maternal continuum of care and its determinants is critical to improve maternal and child health interventions. However, none of the previous studies had explored the spatial distribution of zero utilization for maternal continuum of care. Besides, previous studies were assessed the spatial variation of maternal continuum of care based on Enumeration areas. This study, on the other hand, looked at zonal-based spatial variation, which works by aggregating all of the Enumeration areas that belongs to a specific Zone. Furthermore, previous spatial studies did not used spatial modeling to identify the spatial relationships between maternal continuum of care and spatial related factors affecting it. Thus, this study was aimed to explore geographical variation and predictors of zero utilization for a standard maternal continuum of care among women in Ethiopia.

## Methods

### Study setting

**Fig. 1:**
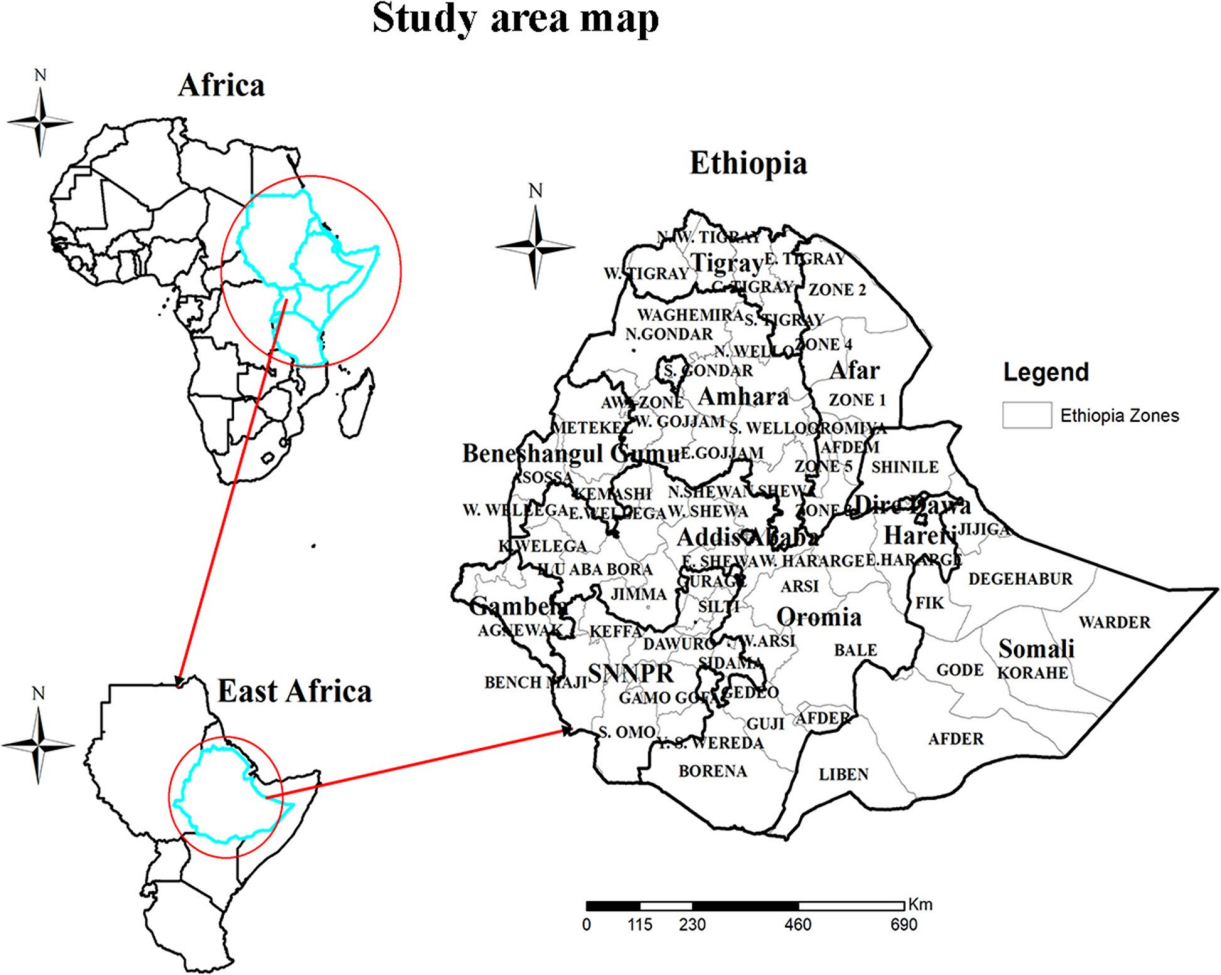
Study area map

The study was conducted in Ethiopia which is found in the horn of Africa. It has a geographical diversity ranging from 4550 m above sea level and 110 m below sea level. Ethiopia has nine administrative regions and two City administrations divided into 817 districts and 16,253 kebeles (Fig. 1). This study used the 2016 EDHS dataset conducted from January 18 to June 27, 2016 [28].

### Population

All women who gave birth five years preceding the data collection period of the 2016 EDHS was considered as a source population, all women who gave birth five years preceding the data collection period of the 2016 EDHS in the selected Enumeration areas was designated a study population. A total of 4178 mothers who full fill the inclusion criteria were considered for the entire analysis. To select study participants a stratified, two-stage cluster sampling technique was employed. Six hundred forty-five Enumeration areas with latitude and longitude coordinates was used for 2016 EDHS. Of these 23 Enumeration areas had no latitude and longitude coordinates. The missed location data may affect the true estimates of the analysis. Location data values were shifted 1-2 km for urban and 5 km for rural areas for data confidentiality reasons. The location data were accessed through the web page of measure Demographic and Health Survey (DHS) Program.

### Study variables

The outcome variable (zero utilization for maternal continuum of care) was measured dichotomously “Yes/No). If a woman had not utilized any component of a standard maternal continuum of care (four or more ANC visits, delivery assisted by SBA, and PNC within 48 h), they are rated as zero utilization for maternal continuum of care. Socio-demographic factors: mothers age, mothers and fathers’ educational level, marital status, mothers and fathers’ employment status, sex of household head, wealth index, occupation, religion, relationship with household head, residence, region, and distance from a health facility; social media: newspaper reading habit, listening radio, having mobile phone and listening television were independent variables.

### Data management and analysis

The data is obtained from the Measure demographic and health survey (DHS) website and data extraction, data weighting, data cleaning, and recoding were performed using STATA version 16.0 Software. The sample distribution to different regions, as well as urban and rural areas, was not proportional in the EDHS dataset. To account for disproportionate sampling and non-response, sample weights was applied to the data to estimate proportions and frequencies. A full clarification of the weighting procedure was explained in the 2016 EDHS report. Descriptive and summary statistics such as frequency table and cross-tabulation were executed.

### Spatial analysis

The Global Moran’s I statistic was held to assess the pattern of zero utilization for maternal continuum of care whether it was dispersed, clustered, or randomly distributed in the study area. Global Moran’s I analysis enables to detect the presence of correlation among non-utilization of maternal continuum of care. This could enable to identify whether there is a correlation among non-utilization of maternal continuum of care or not. Details about spatial autocorrelation is published everywhere [29, 30]. Local Moran’s I measure positively associated clusters and outliers. Furthermore, Local Moran’s I analysis detects outliers of the cluster which is impossible to detect using Getis-Ord analysis and Hot Spot as well as cold spot zones in order to make sure that the consistency of findings by Gettis-Ord analysis [31–33].

### Gettis-Ord Gi* statistics

Gettis-ord Gi* statistics was calculated to measure the difference of spatial autocorrelation in the study location for each area. Gettis-ord Gi* statistics could identify hot spot areas for zero utilization of maternal continuum of care. The z-score was calculated to confirm the statistical significance of clustering and the *p*-value was calculated to determine the significance at *p*-value< 0.05 with 95% CI. If the z-score is less than − 1.96 it is declared as a cold spot and if it is greater than + 1.96 it is declared as hotspot areas [33–36].

### Spatial scan statistical analysis

Spatial scan statistics tests the presence of statistically significant spatial clusters by Sat Scan version 9.6 software. SaT Scan analysis further intensifies the finding which detected by Gettis-Ord analysis which is strong to detect hot spot regions and enables to reports confirmatory findings for the analysis. A woman who had zero utilization for maternal continuum of care was considered as cases and a woman who utilized at least one component of a standard maternal continuum of care was considered as controls [37, 38].

Spatial cluster size < 25% of the population was used, as a higher boundary. The primary and secondary clusters were identified through *p*-values. The probability of false inclusion and exclusion of clusters is limitation of SaT Scan. Details about spatial scan statistical determination is published everywhere [33, 36–39].

### Spatial regression analysis

Spatial regression has both local and global analysis techniques [40–42]. Accordingly, the ordinary least square model is handled first and then geographically weighted regression analysis was executed [43–45]. Assumptions were checked with the respective tests. Koenker Bp test was executed to check the model whether geographically weighted regression analysis could be executed or not. GWR version 4 software was used to execute Geographically weighted regression. The six checks recommended for spatial regression analysis was also checked [33, 46, 47]. Variables that have a *p*-value less than 0.05 with a 95 Confidence interval was selected and described based on their coefficients.

### Ethical consideration

Permission to use the dataset has been granted by the measure DHS program through legal registration. Data from the EDHS (2016) was used, which is available on the Measure DHS website (www.measuredhs.com) [48]. Accordingly, the investigators had granted permission to use the dataset on the Measure DHS website about the geographical variation of zero utilization for maternal continuum of care among women in Ethiopia in September 2020.

## Results

### Socio-demographic characteristics of respondents

A total of 4178 mothers were included in this study. About, 2526 (60.46%) of respondents had no formal education and the majority of respondents, 3673 (87.9%) were rural residents (Table 1).

**Table 1:** sociodemographic characteristics of respondents in 2016 EDHS data for zero continuum of care for maternal health care service utilization

Variables	Frequency	Percentage
Age of the mother (in years)		
15–24	1234	29.54
25–34	2116	50.65
35–49	828	19.81
Mother’s educational level		
No education	2526	60.46
Primary education	1282	30.68
Secondary education /above	370	8.86
Mother’s marital status		
Married	248	5.94
Unmarried	3930	94.06
Sex of household head		
Male	3610	86.41
Female	568	13.59
Mother’s religion		
Orthodox	1426	34.13
Catholic	40	0.95
Protestant	855	20.46
Muslim	1747	41.83
Others	110	2.63
Mother’s employment status		
Employed	1521	36.41
Not employed	2657	63.59
Residence		
Urban	505	12.09
Rural	3673	87.91
Region		
Tigray	305	7.30
Afar	42	1.01
Amhara	767	18.37
Oromo	1857	44.47
Somali	177	4.23
Benishangul-Gumuz	44	1.04
SNNPR	844	20.19
Gambela	10	0.24
Harari	10	0.24
Dire Dawa	17	0.41
Addis Ababa	105	2.51
Wealth status		
Poor	1885	45.12
Middle	867	20.75
Rich	1426	34.13
Father’s educational level		
No education	1810	45.50
Primary	1595	40.09
Secondary/above	573	14.41
Father’s employment status		
Employed	3151	79.22
Not employed	827	78
Distance to the health facility		
Big problem	3773	90
Not big problem	405	10
Has telephone		
Yes	56	1.33
No	4122	98.67
Read magazine		
Yes	297	7.11
No	3881	92.89
Listen to radio		
Yes	1158	27.72
No	3020	72.28
Watching television		
Yes	794	19.00
No	3384	81.00
Relationship to the household head		
Head	399	9.55
Wife	3323	79.55
Daughter	379	9.06
Others	77	1.84

### Zero utilization for maternal continuum of care

**Fig. 2:**
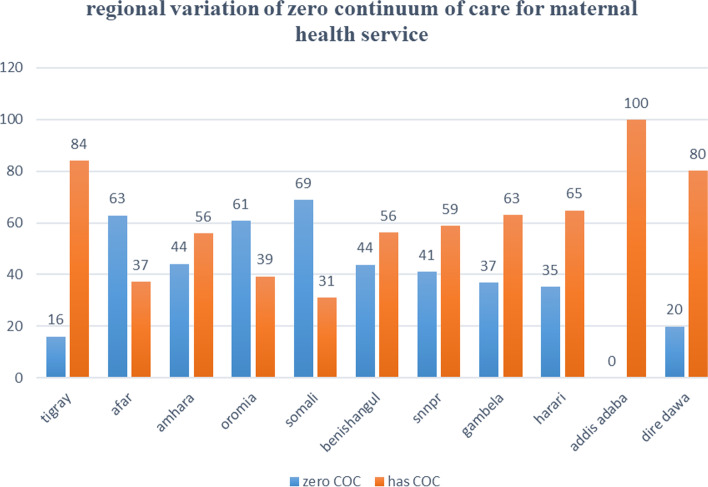
Regional variation of zero utilization for maternal continuum of care in Ethiopia

The proportion of mothers who had zero utilization for maternal continuum of care in Ethiopia was 2039 (48.8%) (95% CI: 47.3–50.4). Zero utilization of maternal continuum of care was lowest in Addis Ababa (0%) and highest in the Somali region (69%) (Fig. 2).

### Spatial variation of zero utilization for maternal continuum of care

**Fig. 3:**
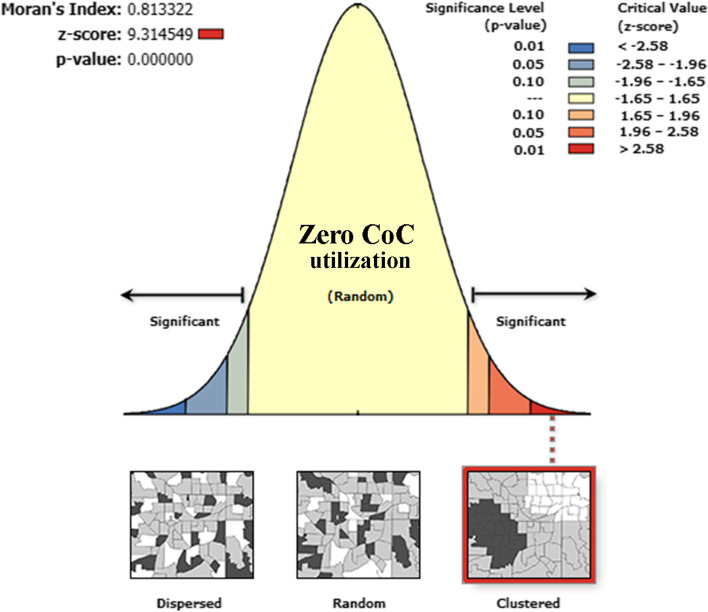
Spatial autocorrelation of zero utilization for maternal continuum of care in Ethiopia

**Fig. 4:**
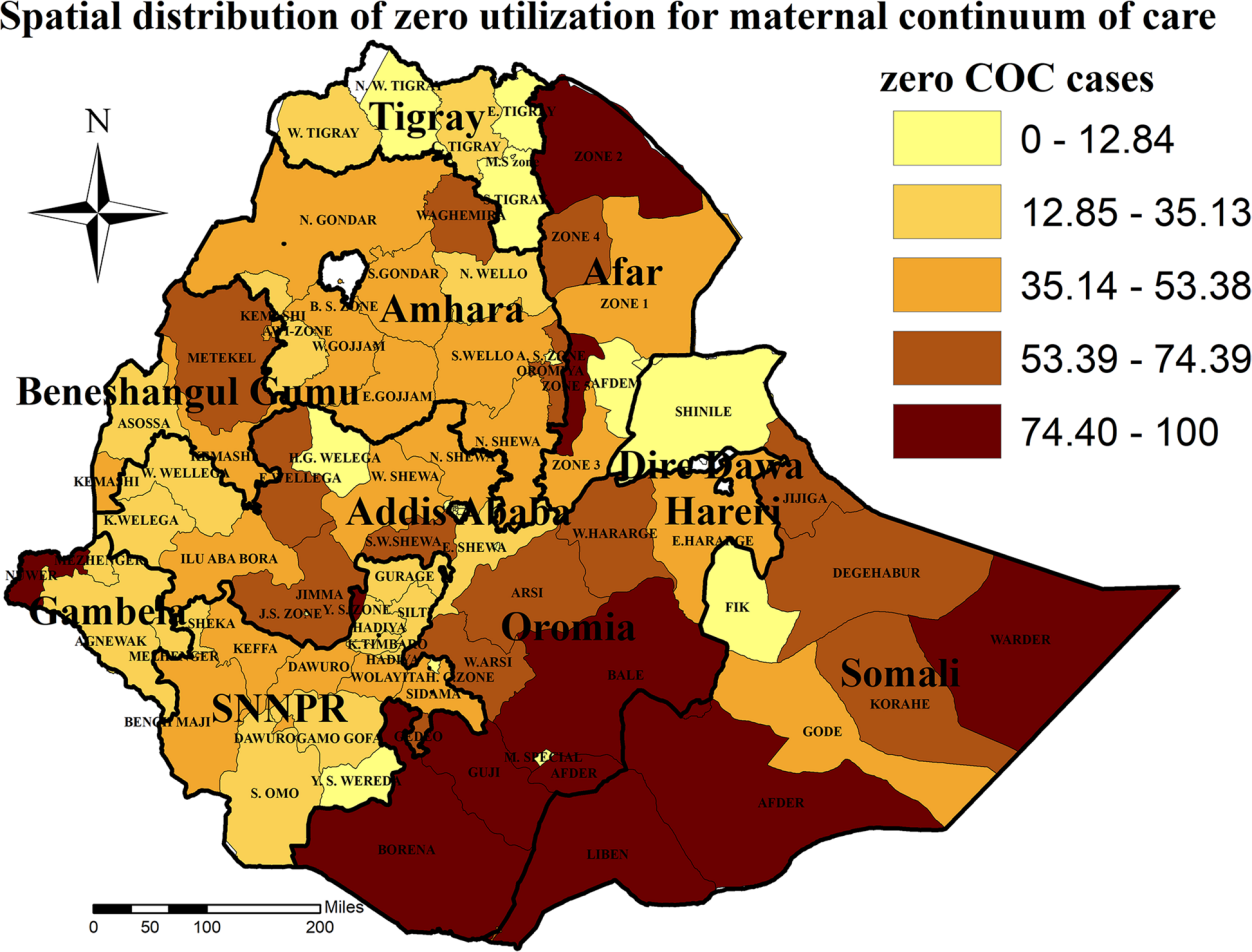
Spatial distribution of zero utilization for maternal continuum of care in Ethiopia

The spatial distribution of zero utilization for maternal continuum of care was clustered at zonal level. Hence, the global Moran’s I index value was 0.8133322 (*p*-value < 0.001), which shows statistically significant clustering (Fig. 3). The highest distribution of zero utilization for maternal continuum of care was found in Zone 2 of Afar region, Afder, Liben, and Warder Zones of Somali region, Bale, Guji and Borena, Zones of Oromia region, and Neuer zone of Gambella region (Fig. 4).

### Hot spot and cold spot zones for zero utilization of maternal continuum of care

**Fig. 5:**
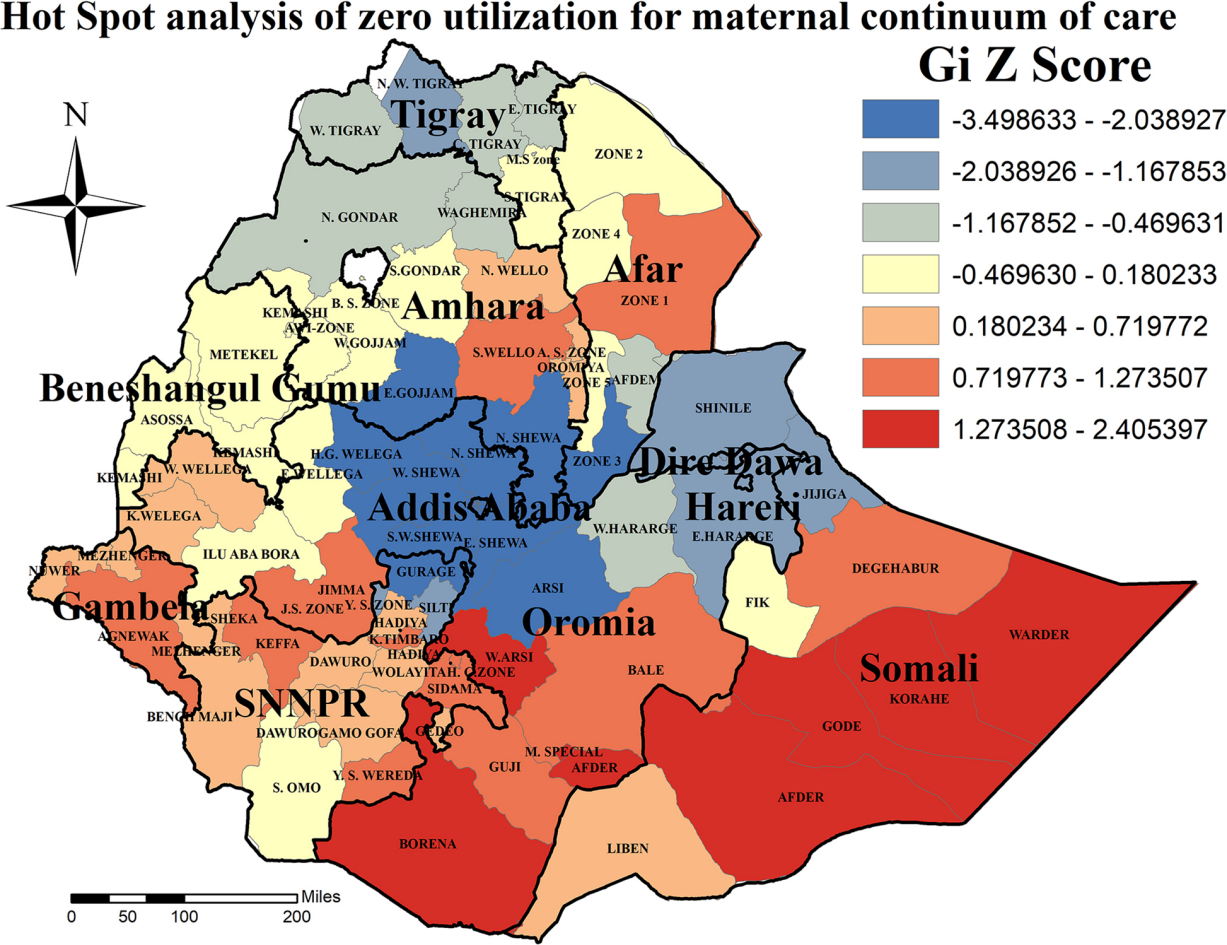
Hot spot analysis of zero utilization for maternal continuum of care in Ethiopia

Hot spot analysis enables the detection of either high or low statistically significant coverage zones to zero utilization of maternal continuum of care. Accordingly, hot spot (high risk) regions for zero utilization of maternal continuum of care was detected in Afder, Warder, Korahe and Gode Zones of Somali region, and West Arsi Zones of Oromia region. On the other hand, North Shewa and East Gojam Zones of Amhara region, Zone 3 of Afar region, Gurage zone of SNNPR region and East Shewa, West Shewa, North Shewa, Arsi, Zones of Oromia region were statistically significant cold spot Zones (Fig. 5).

### Cluster and outliers of zero utilization for maternal continuum of care

**Fig. 6:**
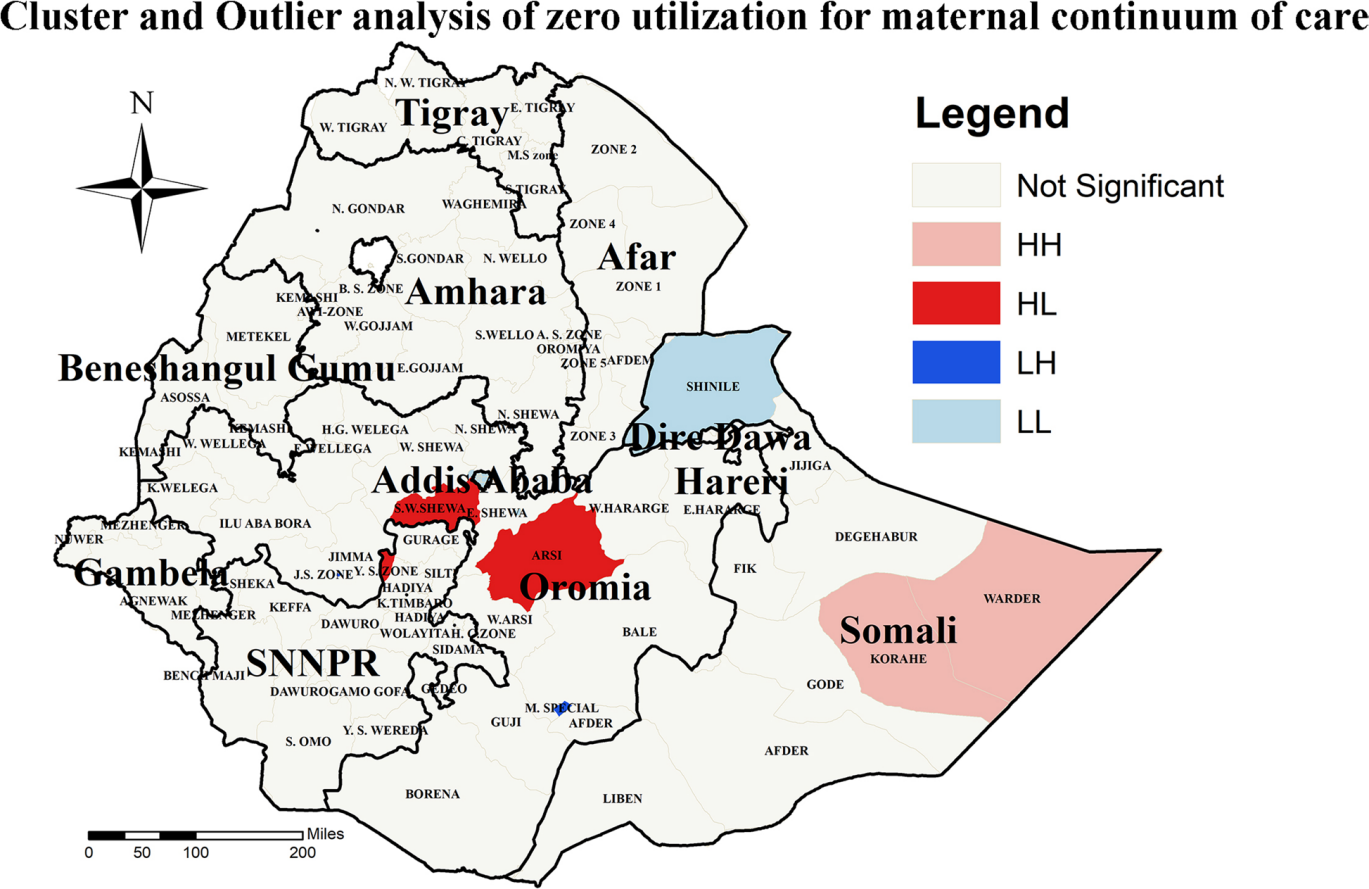
Cluster and outlier analysis of zero utilization for maternal continuum of care in Ethiopia

Cluster and outlier analysis results revealed that there was a significant outlier of zero utilization for maternal continuum of care. Accordingly, high outliers for zero utilization of maternal continuum of care were detected in East Shewa and Arsi zone of Oromia region (Fig. 6).

### Spatial clustering of zero utilization for maternal continuum of care

Spatial scan statistics identified primary and secondary clusters of zero utilization of maternal continuums of care using the maximum spatial circular windows ≤25% of the total population. Accordingly, spatial scan statistics identified (one primary and four secondary clusters) for zero utilization of maternal continuum of care.

**Fig. 7:**
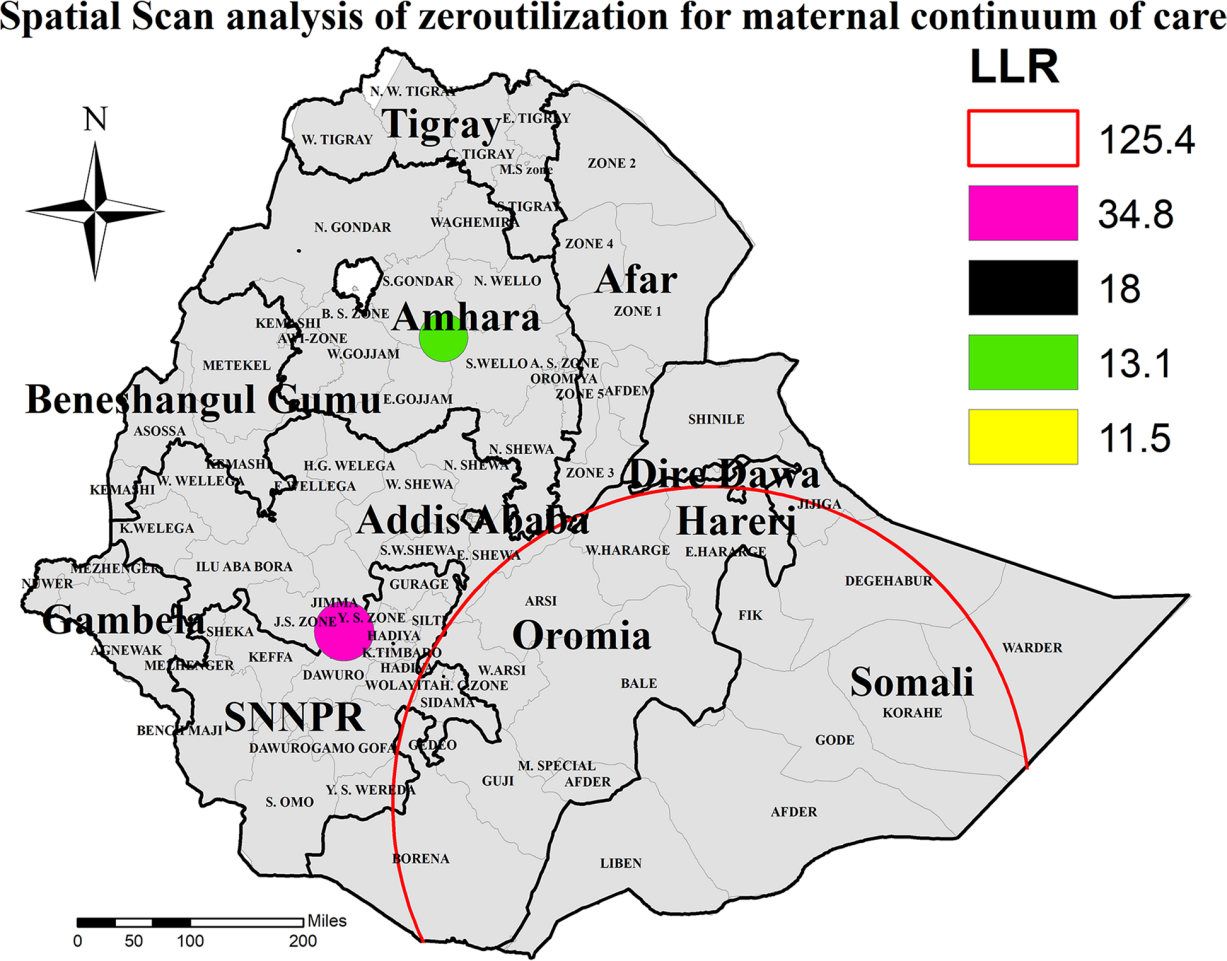
Spatial scan statistics analysis of zero utilization for maternal continuum of care in Ethiopia

The primary cluster for zero utilization of maternal continuum of care (LLR = 125.4, *P* < 0.001) was centered at (5.330795 N, 41.837597 E) with a 451.29 km radius and has a relative risk (RR) of 1.65. It incorporates Degahbur, Korahe, Gode, Warder, Liben, Jijiga, Afder and Fik Zones of Somali region, and East Harrerge, West Harrerge, Arsi, West Arsi, Bale, Guji, and Borena Zones of Oromia region (Table 2, Fig. 7). The finding is interpreted as a woman within the ring is 1.65 times to have zero utilization of maternal continuum of care when compared to a woman away from the ring.

**Table 2:** Summary of sat scan result for zero continuum of care for maternal health service utilization using 2016 EDHS data

Variable	Detected clusters	Coordinate /radius	Population	Cases	RR	LLR
**Zero COC**	Primary cluster**	(5.330795 N, 41.837597 E) / 451.29 km	1383	914	1.65	125.4
	Secondary cluster 1**	(7.557240 N, 37.132572 E) / 42.14 km	96	85	1.85	34.8
	Secondary cluster 2**	(8.411698 N, 38.366035 E) / 0 km	25	25	2.06	18
	Secondary cluster 3**	(11.287790 N, 38.406887 E) / 34.02 km	24	23	1.97	13.1
	Secondary cluster 4**	(10.455377 N, 38.827587 E) / 0 km	16	16	2.06	11.5

### Spatial regression analysis for the determinants of zero utilization of maternal continuum of care

#### Ordinary least square

**Fig. 8:**
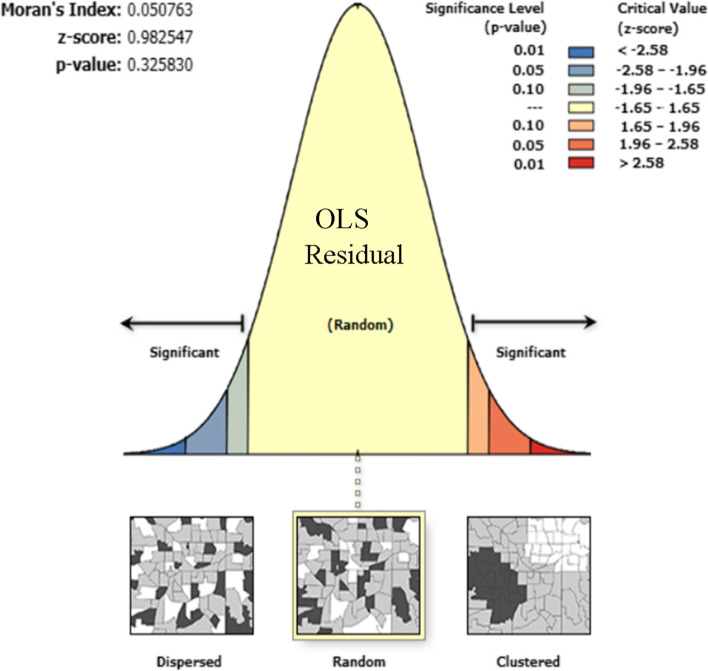
Spatial auto correlation of residuals in ordinary least square analysis

Outputs from the spatial regression analysis revealed that residuals of spatial relationships are uncorrelated and there was no multi-collinearity among explanatory variables (Table 3, Table 4, and Fig. 8). In ordinary least square analysis: poor wealth index, uneducated mothers, and mothers who declare distance as a big problem were factors significantly associated with zero continuum of care for maternal health care service utilization. Respondents who had poor wealth index, uneducated, and who declare distance as a big problem could increase zero utilization for maternal continuum of care by 0.24, 0.27, and 0.1 times (Table 2).

**Table 3:** summary result of ordinary least square (global GWR) coefficients for zero COC for maternal health care service utilization using 2016 EDHS data

Variables	Coefficient	Probability	Robust Probability	Variance inflation factor
Intercept	20.87	0.0003	0.0000	
Poor wealth index	0.24	0.0000	0.0000	1.5
Unmarried mothers	−0.17	0.0662	0.0752	1.5
Uneducated Mothers	0.27	0.0000	0.00000	1.4
Distance is a big problem	0.1	0.02967	0.032763	1.9
Uneducated fathers	0.05	0.5547	0.501683	2
Male household head	−0.04	0.22703	0.21521	2.3

**Table 4:** A summary result of ordinary least square (global GWR) diagnostics for zero COC for maternal health care service utilization using 2016 EDHS data

Diagnostics criteria	Magnitude	*p*-value
AICc	3269.2	
R squared	0.302	
Adjusted R squared	0.3	
Joint f statistics	27	0.0000*
Joint wald statistics	223.6	0.0000*
Koenker (Bp) statistics	15	0.016*
Jareque-Bera statistics	2.22	0.3297

#### Geographically weighted regression

**Fig. 9:**
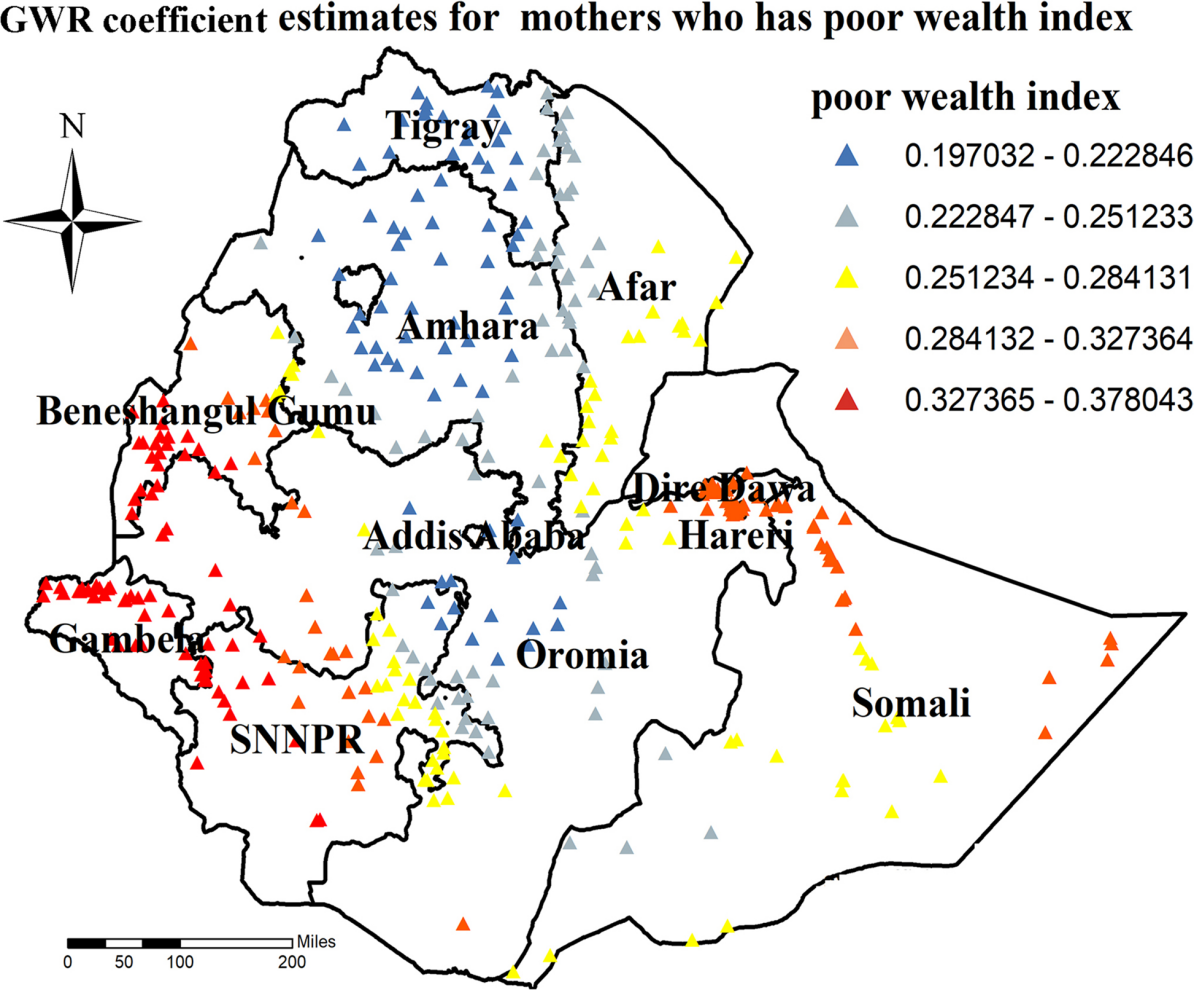
GWR coefficient estimates for poor wealth index predictor of zero utilization for maternal continuum of care in Ethiopia

The result of geographically weighted regression analysis has identified different variable coefficients through different geographical location. Accordingly, higher coefficients of poor wealth index were detected in all parts of Gamballa, South-Western Benshangul-gumz, South-Eastern Benshangul-gumz, North Western parts of SNNPR, Harrari region, and Diredawa city administrative (Fig. 9).

**Fig. 10:**
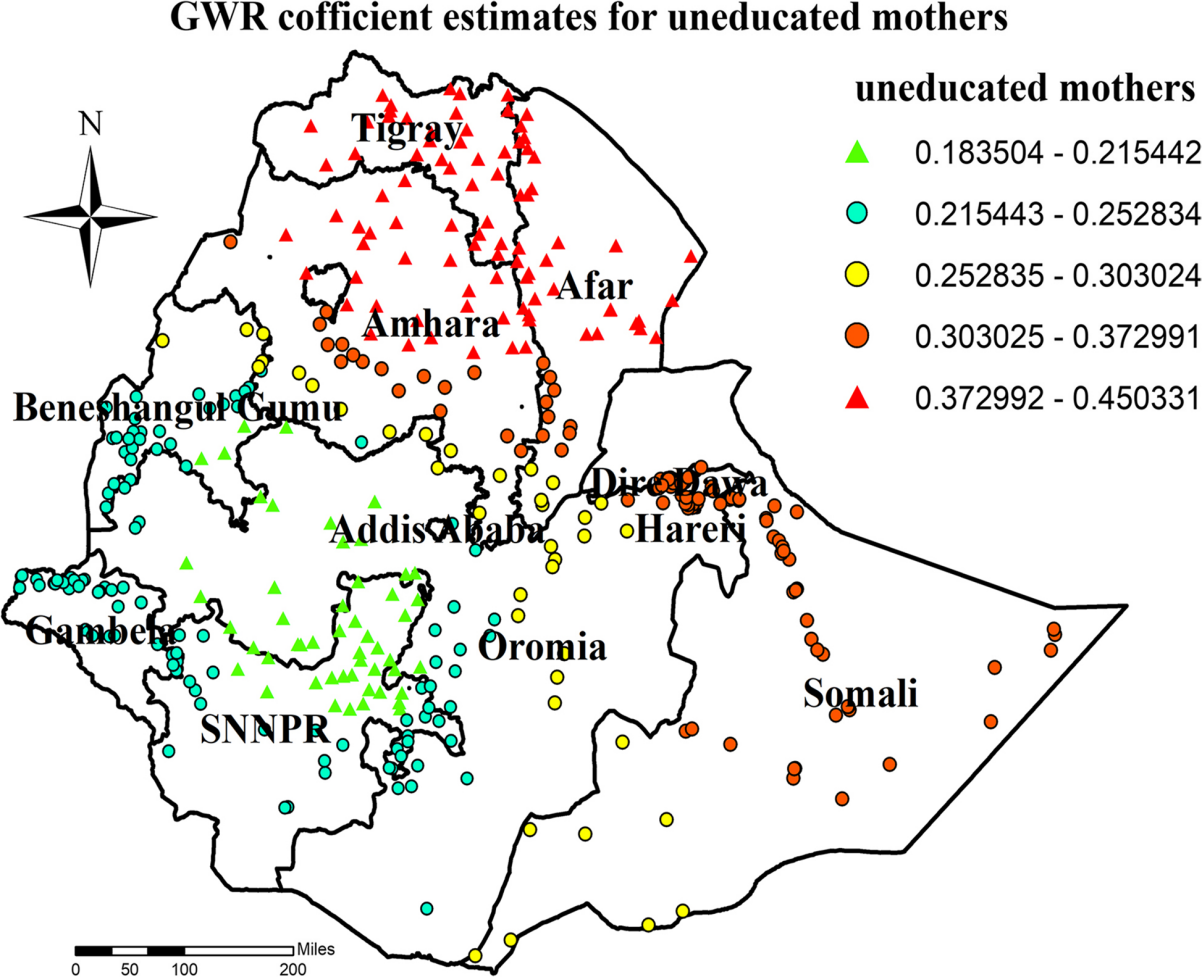
GWR coefficient estimates for uneducated mother predictor of zero utilization for maternal continuum of care in Ethiopia

**Fig. 11:**
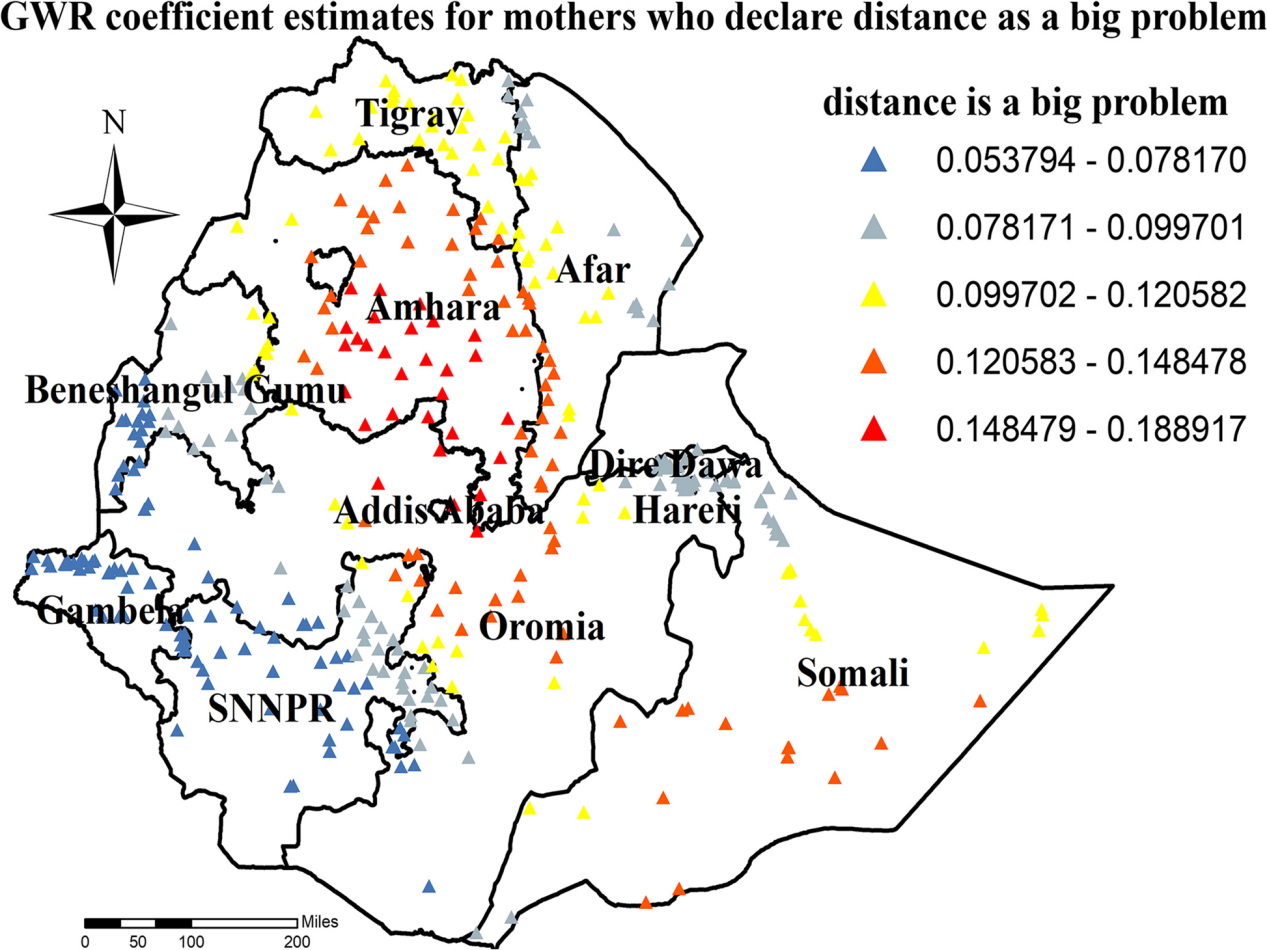
GWR coefficient estimates for mother who declare distance as a big problem predictor of zero utilization for maternal continuum of care in Ethiopia

Similarly, higher coefficients for mothers who are not educated were detected in all parts of Tigray, Amhara, Afar and Somali region (Fig. 10). Furthermore, higher coefficients for mothers who declare distance as a big problem were detected in most parts of the Amhara, some parts of Somali, and Central Oromia region (Fig. 11).

## Discussion

Eventhough too many initiatives have been held in Ethiopia in recent years to improve maternal continuum care use, utilization of maternal continuum care remains a major challenge. In this study, The proportion of mothers who had not utilized any components of a standard maternal continuum of care was 48.8% (95% CI: 47.3–50.4). The use of a standard maternal continuum of care also varied between Ethiopian administrative regional states. Various studies on non-utilization of maternal continuum of care in Ethiopia and abroad have found a considerable rate of non-utilization.

A study conducted in South Sudan based on 2010 household survey describes 58% women did not utilized ANC service [49]. In addition, a study conducted in Rwanda revealed that about 54% of pregnant women did not make the recommended four visits to ANC during pregnancy [50]. Similarly, different studies conducted in Ethiopia [51–54] and abroad [55–59] revealed that, majority of pregnant women were assisted by non-skilled birth attendant and deliver their baby at home. On the other hand, a study conducted in Western Ethiopia revealed that around 90% women could not utilized appropriate postnatal care according to WHO guidelines [60]. Similarly, a study conducted in Nigeria revealed that about 75.8% of the respondents did not attend PNC services [61] and another study conducted in Nigeria showed that 96% of the mothers did not utilize postnatal care services [62]. In addition, a study conducted in Pakistan showed that (37%) did not utilize PNC check-ups in the first 2 days after delivery [63].

Geographic location, socio-demographics, and health inequities, as well as economic concerns among diverse groups, may all play a role in the underutilization of maternal health services. Furthermore, non-utilization of maternal continuum of care may be influenced by societal culture, attitudes, and knowledge in different parts of Ethiopia.

Different studies in Ethiopia have reported that, women who had at least one ANC visit ranges from 54 to 76% [27, 64–70] which is contrary to the current finding. This disparity can be explained by the fact that this study looked at zero utilization of maternal continuum of care based on a standard sated for maternal health service utilization. However, previous studies were assessed for at least one ANC visit of a women. On the other hand, this study had evaluated non utilization of any component of a standard maternal continuum of care. Therefore, the outcome variable of our study has some differences from previous studies.

Different studies in Ethiopia [71, 72] and abroad [73–78] also reported low completion of maternal continuum of care. There are also studies that reported relatively moderate completion of maternal continuum of care [77, 79, 80]. On the other hand, there are studies that reported relatively high completion of maternal continuum of care [81, 82].

This low utilization of maternal continuum of care may be due to regional differences in terms of health services accessibility, health service quality, compliance of the care among women, delivery of compassionate care among health care providers, and socioeconomics status of respondents. Furthermore, low implementation of preconception care is a contributing factor for low completion of maternal continuum of care.

The finding of the current study showed that zero utilization of maternal continuum of care was clustered spatially at zonal level. Spatial autocorrelation analysis detected the presence of correlation among non-utilization of maternal continuum of care. Getis-Ord spatial analysis detected that hot spot and cold spot Zones of zero utilization for maternal continuum of care in Ethiopia. In addition, hot spot zones identified using Getis-Ord analysis was confirmed using saT Scan analysis. Furthermore, cluster and outlier analysis was determined the presence of outlier Zones for zero utilization of maternal continuum of care. Overall the spatial analysis had confirmed the presence of hot spot zones for zero utilization of maternal continuum of care and had intensified this finding through different geostatistical techenique.

Different studies also support the presence of geographical variation for maternal continuum of care utilization. A study conducted on geographic differences in maternal and child health care utilization in four regions of Ethiopian, revealed that some of the districts in the Tigray region showed that clustering of high levels of full immunization coverage. In contrast, in the Oromia region there were districts with indications of clustered with low coverage [83]. A study conducted on maternal healthcare service use in Jimma Zone, revealed that four kebeles in Bula Wajo primary health care unit and one in Adere Dika has low antenatal care utilization. Sinkulle and Dogoso in Bula Wajo primary health care unit had low institutional delivery and Bula Wajo primary health care unit, Kora Wacho in Seka Chekorsa had low utilization of postnatal care [84]. Different studies also support the presence of geographical variation of maternal continuum of care utilization [22, 24–26, 83, 85].

The probable explanation of this spatial variation might be a geographical variation that exists in the country. Consequently, infrastructure differences like electricity, road, water, the distribution of health care professionals, and health facilities across regions. In addition, different regions have distinct cultures, socio-demographic features of respondents, attitudes, and understanding of the society when it comes to use maternal continuum of care. Moreover, health service quality, compliance of the care among women, delivery of compassionate care among health care providers could also inhibit the involvement of women in the continuum of care. Overall this form of difference in Ethiopia could bring variations of zero utilization for maternal continuum of care across different parts of the country.

In spatial regression analysis, different statistically significant predictors of zero utilization for maternal continuum of care were identified. Accordingly, poor wealth index, uneducated mothers, and mothers who declare distance as a big problem were factors significantly associated with zero utilization of maternal continuum of care. Different studies conducted in Ethiopia and abroad also revealed that, there is a considerable significant difference of predictors for a certain outcome variable across a geographical area [54, 86–89].

Mothers who are not educated were positively correlated with zero utilization of maternal continuum of care and its coefficients significantly varied across regions of Ethiopia. Those areas which have higher coefficients for uneducated mothers also have hot spots of zero utilization for maternal continuum of care. Education is one of the most important strategies for bolstering and gaining acceptance of scientific arguments for maternal health-care service consumption, as well as for resolving issues related to maternal continuity of care. Respondents with a higher level of education were more likely to comprehend and persuaded by health care providers than those with a lower level of education. Furthermore, an educated mother might inquire about anything that perplexed her during service delivery or advocacy. As a result, illiterate moms’ chances of being involved in any aspect of maternal continuum of care may be reduced.

Similarly, respondents who stated distance from health facilities as a major issue were shown to be positively associated with zero utilization of maternal continuum of care. Those areas which have higher coefficients for mothers who declare distance from health facility as a big problem also has hot spots of zero utilization for maternal continuum of care. The primary impediment to receiving maternal health care is distance. Because transportation access and transportation fees may be a problem for women who live distant from health services. Furthermore, individuals who live far away from health institutions may not have access to maternal health information. This may make it difficult for mothers to use any of the components of the continuum of care. Furthermore, mothers who have poor wealth index was positively correlated with zero utilization for maternal continuum of care. Those areas which have higher coefficients of poor wealth index also have hot spots of zero utilization for maternal continuum of care. This might be mothers could be challenged to address issues that arise from the family since, they could be torched by preliminary activities to lead their family. In addition, mothers could handle different occasions of family concern other than men. Thus, they may encounter difficulty in handling issues related to maternal continuum of care.

### Strength and limitation of the study

#### Strength

The finding of this study can be applied to all Ethiopian women who give birth. In addition, the application of different geostatistical analyses assisted in the detection of similar and statistically significant high-risk clusters and enables to triangulate findings through different geostatistical analyses. Furthermore, the use of geographic weighted regression analysis could bring additional qualities in order to identify predictors of maternal continuum of care at each specific geographic area. Lastly spatial clusters detected in this study is zonal based clusters in light of previous findings.

#### Limitation

For data confidentiality reasons, location data values were shifted 1–2 km for urban Enumeration areas and 5 km for rural Enumeration areas and this might not provide the exact case locations. The missing data may have an impact on the true estimates of the analyses.

### Implications of the study

This study revealed unique hotspot zones and characteristics that have a substantial impact on non-utilization of maternal continuum of care. Even while there is evidence that factors such as regional features have an impact on non-utilization of maternal continuum of care, it is difficult to pinpoint specific hot spot areas for which this study could provide a solution. The findings of this study could be essential for mother and child health planning at the zonal and regional levels. Regional patterns of variables utilized in this study suggest that zonal targeted activities should be strengthened and continued. Finally, the identification of determining elements based on geographic area could imply that distinct strategical interventions could be conducted in different hot spot Zones.

### Recommendations

The Ethiopian federal ministry of health and stakeholders working on maternity and child health programs could hold a geographic-based intervention to curb the high prevalence of zero continuum of care utilization. The Ethiopian federal ministry of health, in collaboration with regional health offices in Somalia and Oromia, could invest their maximum effort to reduce zero utilization of maternal continuum of care hot spots recognized across Somali and Oromia regions. In addition, stakeholders who are working on maternal and child health programs could support different strategic interventions designated to decrease zero maternal continuum of care utilization.

Additionally, improving the lifestyle of the community and education in areas identified as hot spot for zero maternal continuum of care utilization should be a strategic tool to decrease zero maternal continuum of care utilization. Accordingly, Somali region and Oromia region should focused education based interventions for areas identified as hot spot for zero maternal continuum of care utilization. Furthermore, additional health facilities could be built for peoples who are far away from the health facility based on the standard distance in order to increase the accesebility of health facilities for maternal health service uptake. Similarly different strategies could implmented to improve the lifestyle and economy of the society. Since economicaly secured women could work for quality of life.

## Conclusion

This study revealed that five out of ten women could not utilize any components of maternal continuum of care based on the standard. This study also showed that the distribution of zero utilization for maternl continuum of care was clustered at zonal level in Ethiopia. zero utilization for maternl continuum of care had a geographical variation across regions of Ethiopia. Hot spot (high risk) regions for zero utilization for maternal continuum of care were detected in Somali region (Afder, Warder, Korahe and Gode Zones), and West Arsi Zones of Oromia region.

In geographical weighted regression analysis, different statistically significant predictors for zero utilization of maternal continuum of care were identified. Accordingly, poor wealth index, uneducated mothers, and mothers who declare distance as a big problem were factors significantly associated with zero utilization for maternal continuum of care.

## Data Availability

All relevant data are in the manuscript. However, the minimal data underlying all the findings in the manuscript will be available upon request. EDHS (2016) data was used which is available on the public domain through the Measure DHS website (www.measuredhs.com) [48].

## Abbreviations

COC: A continuum of care for maternal health care service utilization
RMNCH: Reproductive, maternal, neonatal, and child health
SSA: Sub-Saharan Africa countries
DHS: Demographic and Health Survey
ANC: Antenatal care
SBA: Skilled birth Attendant
PNC: Postnatal care
EDHS: Ethiopian Demographic and Health Survey
ICF: International Classification of Functioning, Disability, and Health
LLR: Log-likelihood ratio
SD: Standard Deviation
UNICEF: United Nation International Children’s Fund
WHO: World Health Organization
EFY: Ethiopian Fiscal Year
FMOH: Federal Ministry of Health
GWR: Geographic Weighted Regression
OLS: Ordinary Least Square
